# The stability of perennial grasses mediates the negative impacts of long-term warming and increasing precipitation on community stability in a desert steppe

**DOI:** 10.3389/fpls.2023.1235510

**Published:** 2023-07-27

**Authors:** Guangyi Lv, Mengting He, Chengjie Wang, Zhanyi Wang

**Affiliations:** Key Laboratory of Grassland Resources of the Ministry of Education/College of Grassland, Resources and Environment, Inner Mongolia Agricultural University, Hohhot, China

**Keywords:** climate change, desert steppe, plant functional groups, species diversity, community stability

## Abstract

**Background:**

Desert steppe, as an ecotone between desert and grassland, has few species and is sensitive to climate change. Climate change alters species diversity and the stability of functional groups, which may positively or negatively affect community stability. However, the response of plant community stability in the desert steppe to experimental warming and increasing precipitation remains largely unexplored.

**Methods:**

In a factorial experiment of warming and increasing precipitation for five to seven years (ambient precipitation (P0), ambient precipitation increased by 25% and 50% (P1 and P2), ambient temperature (W0), ambient temperature increased by 2°C and 4°C (W1 and W2)), we estimated the importance value (IV) of four functional groups (perennial grasses, semi-shrubs, perennial forbs and annual herbs), species diversity and community stability.

**Results:**

Compared to W0P0, the IV of perennial grasses was reduced by 37.66% in W2P2, whereas the IV of perennial forbs increased by 48.96%. Although increasing precipitation and experimental warming significantly altered species composition, the effect on species diversity was insignificant (*P* > 0.05). In addition, increasing precipitation and experimental warming had a significant negative impact on community stability. The stability of perennial grasses significantly explained community stability.

**Conclusion:**

Our results suggest that the small number of species in desert steppe limits the contribution of species diversity to regulating community stability. By contrast, maintaining high stability of perennial grasses can improve community stability in the desert steppe.

## Introduction

Ecosystem stability is a critical indicator to assess the sustainability of services provided by the ecosystem to humanity (Pimm, 1984), and is regulated by multiple factors (Lehman and Tilman, 2000). Early studies concluded that ecosystem stability depends on biodiversity (Watts, 1973). Complex biodiversity maintains high ecosystem stability (Pimm, 1984). Since the mass ratio theory was proposed (Grime, 1998), numerous studies have verified that functional groups with dominant species have a non-negligible influence on ecosystem stability (Sasaki and Lauenroth, 2011; Yang et al., 2017; Zhou et al., 2020; Li et al., 2021; Ebel et al., 2022). Ecosystem stability is driven by the stability of dominant species if there are a number of dominant species in the plant community (Ma et al., 2017). Therefore, ecosystem stability is jointly driven by multiple factors, and the driving factors may vary between study sites.

The temporal stability of the community is the ability of the community to maintain a stable state in the face of temporal changes in the external environment, and has been the subject of considerable recent research (Chen et al., 2016; Donohue et al., 2016; Zhang et al., 2020; Yang et al., 2021). Similar to ecosystem stability, community stability is influenced by a range of factors. On the one hand, some meta-analyses have suggested that species diversity regulates community stability (Jiang and Pu, 2009; Gross et al., 2014), and that the stability mechanism of species diversity was explained by the overyielding effect, the species asynchrony effect or the portfolio effect (Lehman and Tilman, 2000; Hector et al., 2010; Yang et al., 2011a). On the other hand, recent research has demonstrated that the relative abundance of dominant species has a non-negligible influence on the stability of the community, and that community stability is largely determined by the stability of dominant species (Xu et al., 2015; Zhou et al., 2020; Wu et al., 2022). In addition, the composition of plant functional groups, leaf dry matter content and plant litter also affect community stability (Su et al., 2019; Zhou et al., 2019; Liu et al., 2021; Hou et al., 2022; Ma et al., 2022). Therefore, a variety of factors influence community stability, and all the factors affecting community stability may be altered by climate warming and increasing precipitation. Factors affecting the community stability would be different with grassland types.

The Fifth Assessment Report of the Intergovernmental Panel on Climate Change(IPCC AR5) pointed out that irreversible climate warming and more frequent precipitation will be experienced in mid and high latitudes in the northern hemisphere (Hoegh-Guldberg et al., 2018), which may alter the diversity and composition of species, affect the stability mechanism of plant communities and increase the uncertainty of ecosystems (Wu et al., 2020; Lv et al., 2021). Species diversity, as a combined indicator of species evenness and species richness, can reflect change in community stability (Yang et al., 2011a; Gross et al., 2014; Wang et al., 2021), and has often used to explore the impact of environmental factors on community stability (Yang et al., 2011b; Ren et al., 2017; Zhao et al., 2019). A series of reports have concluded that the composition and diversity of species in grasslands of northern China are regulated by precipitation (Xu and Wang, 2016; Ma et al., 2020). Increasing precipitation improves the richness and evenness of species in the plant community, while warming reduces them (Yang et al., 2011b; Hou et al., 2013). Precipitation and temperature affect the stability of the community by altering species diversity (Zhang et al., 2018). These studies further shown that plant community composition in grasslands is very sensitive to precipitation and temperature. Several studies have found that the importance value of perennial grasses reduces with increasing precipitation and climate warming, whereas the importance value of perennial forbs increases (Yang et al., 2011a; Yang et al., 2011b). Precipitation and temperature affect the community stability of grasslands by altering the structure of plant functional groups (Hou et al., 2022). However, other studies have presented contrasting findings, suggesting that species diversity in desert steppe was not sensitive to temperature and precipitation (Wan et al., 2018), and that the community stability of grasslands in northern China was mainly dependent on the stability of dominant species rather than species diversity (Ma et al., 2017; Wu et al., 2020). Therefore, climate warming and increasing precipitation may affect community stability in northern China’s grasslands by altering species diversity or the stability of dominant species. The purpose of the study is to explorer the mechanism of change of community stability in desert steppe grassland under the future climate warming and increasing precipitation.

Desert steppe, as an ecotone between desert and grassland, located in the east of the Eurasian temperate steppe. Compared with other grassland types, it has low vegetation cover (Angerer et al., 2008). However, the influences of long-term increased precipitation, experimental warming and their interaction on the composition of the plant community and species diversity in the desert steppe remain unclear, and the contributions of the stability of functional groups or species diversity to community stability have not been fully investigated. Given the projected climate changes, there is an urgent need to explore the stabilization mechanism of plant communities in the desert steppe under climate change. Previous research has shown that the desert steppe has few species (Wu et al., 2021), and that the impacts of environmental change on species diversity in the desert steppe was not obvious (Wan et al., 2018). Therefore, we first hypothesized that the influences of long-term increasing precipitation and warming on species diversity and plant community composition in the desert steppe would be insignificant. In addition, perennial grasses are the dominant species in the desert steppe, accounting for the largest proportion of the plant community (Ye et al., 2020; Wu et al., 2021). We further hypothesized that increased precipitation and warming would have a negative effect on community stability, and that community stability would depend on the stability of perennial grasses rather than species diversity.

## Materials and methods

### Study site

The field experiment site is located in Siziwang Banner (at 1445 m above sea level, N41°47′19″, E111°53′45″), Inner Mongolia, northern China. The prevailing temperate monsoon climate in the study site determines that the plant growing season is from May to October (with a peak growing season in mid-August). The average ambient temperature in the growing season was 16.14°C in 2019-2021 (**Figure S1A**), and average natural precipitation was 216.16 mm (**Figure S1C**). Based on the Chinese soil classification, the primary soil type in the study area is a light chestnut soil. With sandy loamy soils, the soils are classified as a Haplic Calcisol according to the FAO soils classification system. **Table S1** shows the basic indicators of soil (0-30 cm) in the study site. The constructive species (the dominant species in the dominant plant layer) of the study site is *Stipa breviflora* Griseb., and the dominant species are *Cleistogenes songorica* Ohwi and *Artemisia frigida* Willd. **Table S2** shows the species classification and the composition of plant communities in the study site (Wu et al., 2020).

### Experimental design

**Figure 1:**
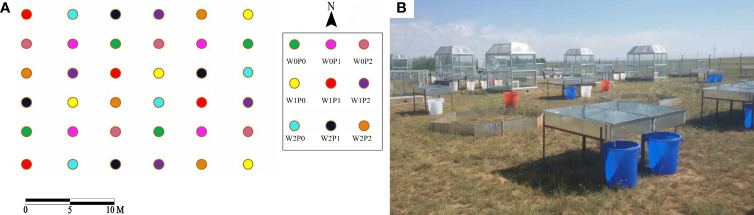
Study site **(A)** and equipment used to experimental warming and increasing precipitation **(B)**. W0, W1, W2, P0, P1 and P2 are ambient temperature, ambient temperature increased by 2°C and 4°C, natural precipitation, natural precipitation increased by 25% and 50%, respectively.

The study used a factorial experiment with climate warming and increasing precipitation designed and established by the Institute of Botany of the Chinese Academy of Sciences in 2014. Before 2014, the experimental site was free grazing grassland. Precipitation and ambient temperature were the two main manipulative factors, and each factor had three levels, i.e., ambient precipitation (P0), ambient precipitation increased by 25% and 50% (P1 and P2), ambient temperature (W0), ambient temperature increased by 2°C and 4°C (W1 and W2). The open-top chamber (OTC) method was used to simulate climate warming. For OTCs, the bottom was a regular hexagon with a side length of 1.5 m, heights of 1 m and 2.3 m (**Figure 1B**), respectively. The impact of OTCs on ambient temperature during the growing season is shown in **Figure S2**. The light transmittance of the OTCs exceeded 95%, and there were two ventilation fans for air circulation. In addition, precipitation was collected by rain interception devices (**Figure 1B**). The areas of the rain interception devices were 25% and 50% of the OTC base area, respectively. The rain interception devices collected natural precipitation into designated buckets during the plant growing season. To achieve increased precipitation, the collected precipitation was manually spread in each OTC after each rain event. The experiment was designed in a randomized block group with nine treatments (i.e., W0P0, W1P0, W2P0, W0P1, W1P1, W2P1, W0P2, W1P2 and W2P2), and each treatment had four replicates, making a total of 36 plots (**Figure 1A**).

### Sample collection and calculation

Experimental samples were collected in mid-August 2019-2021, i.e., after five to seven years of increased precipitation and experimental warming. We described all species in the 1 m × 1 m quadrat in the center of each OTC and recorded the cover, height, density and frequency of all species. For each species, we cut 5-10 plants with similar morphology outside the permanent quadrats and the aboveground biomass of all species was estimated by multiplying the average dry weight of each species by the density of that species in the quadrat. We used formula 1 to estimate the importance value of all species in the quadrats (Curtis and McIntosh, 1951). According to the different life forms of species (**Table S2**), we divided all species into four functional groups (Bai et al., 2004), i.e., perennial grasses (PG), semi-shrubs (SS), perennial forbs (PF), and annual herbs (AH). We summed the importance value of species belonging to the same functional group to obtain the importance value of each functional group. We also calculated indexes of species diversity, community stability, species asynchrony and the stability of the four functional groups using the following formula:


(1) IV=(Hr+Cr+Dr+Fr+Br)/5 

(2) $$H^{\prime }=-\sum_{i=1}^{S}P_{i}\;lnP_{i}$$

(3) $$D=1-\sum_{i=1}^{S}{\left(n_{i}/N\right)}^{2}$$

(4) $$E_{H}=H'/lnS$$

(5) F=S−1/lnN

(6) $$Species\;asynchrony=1-\varphi_{x}=1-\sigma^{2}/{\left(\sum_{i=1}^{S}\sigma_{i}\right)}^{2}$$

(7) Temporal stability of community=µ/σ

where *IV*, *F*, *E^H^*, *D* and *H′* are importance values, Margalef’s index, Pielou’s index, Simpson’s index and Shannon’s index, respectively. *Hr*, *Cr*, *Dr*, *Br* and *Fr* are the relative height, cover, density, biomass and frequency, respectively, which were calculated using Eq. 1-5 in the **supplementary material**. *Pi* is the IV of species *i* in the permanent quadrats, *n_i_* is the number of individuals of species *i*, *N* is the total number of individuals of all species in the permanent quadrats, *S* is the number of species in the permanent quadrats, *φ^x^* is species synchrony, *σ^2^* is the variance of the total number of individuals in the community, *σ^i^* is the standard deviation (s.d.) of the density of species *i* in the permanent quadrats with *S* species, *µ* is the mean density, and *σ* is the s.d. of *µ*.

### Data analysis

We used the general linear model in IBM SPSS Statistics 22 (Armonk, USA) to analyze the influences of warming, increased precipitation and their interactions on the importance value and stability of four functional groups, the index of species diversity and community stability. In addition, Pearson correlation analysis was used to analyze the significance of relationships between species diversity and community stability. We used a general linear model as follows:


(8) y=W+P+W×P

where *y* is the index of species or plant community, *W* is warming, *P* is increased precipitation and × is their interaction.

A structural equation model was executed in AMOS 21.0 (Amos, USA). Firstly, based on the general linear model and correlation analysis, we hypothesized causal relationships between climate change factors (warming and precipitation) and observed variables (species diversity, community stability, species asynchrony and the stability of four functional groups), and constructed a model including all variables and hypothesized relationships. Secondly, we used a maximum likelihood estimation technique to obtain path coefficients, and then eliminated the insignificant paths until we obtained the final model. Finally, we used Chi-square and the root mean square error of approximation to evaluate the fit of the model. We used Origin Pro 2022b (Origin Lab, Massachusetts, USA) to produce the figures and Microsoft Excel 2019 to produce the tables in this paper. Statistical significance, strong significance and extreme significance were expressed as *P*< 0.05, *P*< 0.01 and *P*< 0.001, respectively.

## Results

### Variations in the importance value of four functional groups

**Figure 2:**
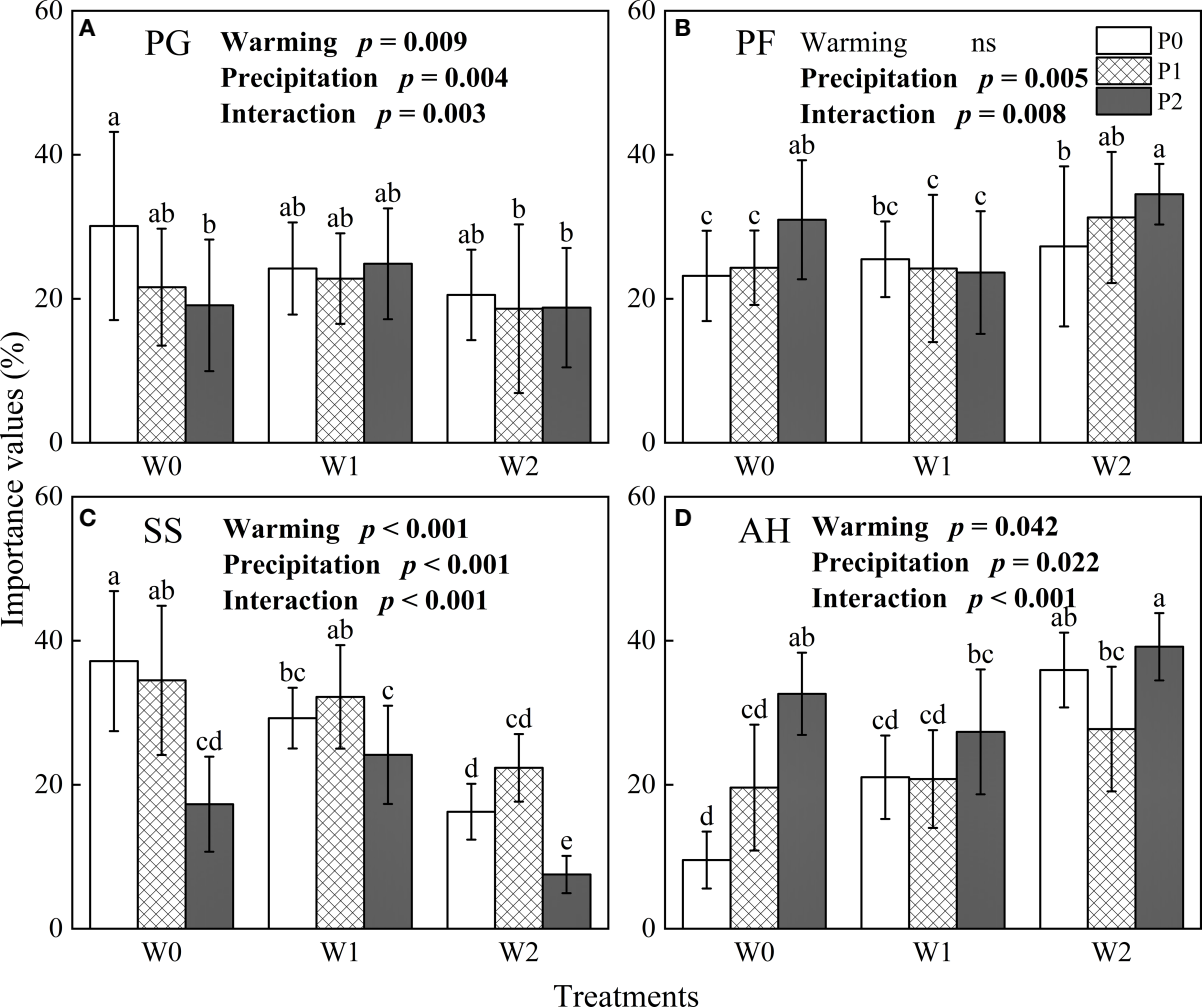
The influences of experimental warming (W), increasing precipitation (P) and their interactions (W×P) on the importance value of four functional groups. **(A)**: perennial grasses, **(B)**: perennial forbs , **(C)**: semi-shrubs and **(D)**: annual herbs. PG, PF, SS, and AH are perennial grasses, perennial forbs, semi-shrubs and annual herbs, respectively. W0, W1, W2, P0, P1 and P2 are ambient temperature, ambient temperature increased by 2°C and 4°C, natural precipitation, natural precipitation increased by 25% and 50%, respectively. The same lowercase letters mean insignificant differences between treatments. “ns” is not significant (*P* > 0.05).

The importance value of four functional *g*roups was significantly affected by increasing precipitation, climate warming and their interaction (*P*< 0.05, **Figure 2**). With increasing temperature and precipitation, the importance values of perennial grasses (PG, W0P0: 30.11 ± 13.07%, W2P2: 18.77 ± 8.29%, mean ± standard deviation) and semi-shrubs (SS, W0P0: 37.17 ± 9.72%, W2P2: 7.53 ± 2.59%) decreased significantly (*P*< 0.05, **Figures 2A, C**), while the importance value of perennial forbs (PF, W0P0: 23.18 ± 6.27%, W2P2: 34.53 ± 4.21%) and annual herbs (AH, W0P0: 9.54 ± 3.95%, W2P2: 39.17 ± 4.68%) increased significantly (*P*< 0.05, **Figures 2B, D**).

### Variations in species diversity

**Figure 3:**
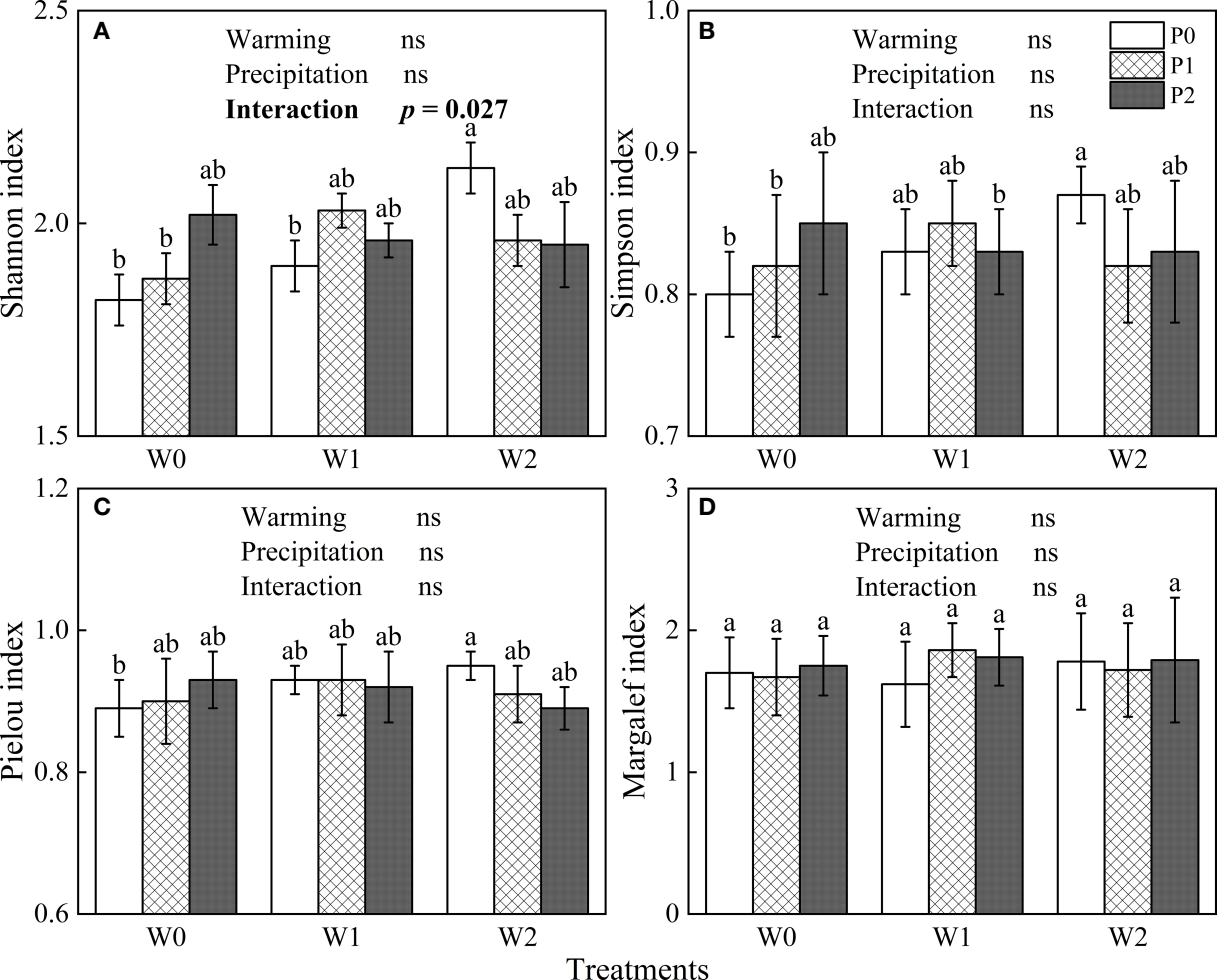
The influences of experimental warming (W), increasing precipitation (P) and their interactions (W×P) on indexes of species diversity. **(A)**: Shannon index, **(B)**: Simpson index, **(C)**: Pielou index, **(D)**: Margalef indexes. W0, W1, W2, P0, P1 and P2 are ambient temperature, ambient temperature increased by 2°C and 4°C, natural precipitation, natural precipitation increased by 25% and 50%, respectively. The same lowercase letters mean insignificant differences between treatments. “ns” is not significant (*P* > 0.05).

The general linear model showed that increased precipitation, experimental warming and their interaction did not significantly affect the Simpson, Pielou or Margalef indexes (*P* > 0.05, **Figures 3B-D**), but the interaction of increased precipitation and warming had a significant effect on the Shannon index (*P*< 0.05, **Figure 3A**). In the ambient temperature treatment, increasing precipitation increased the four species diversity indexes (**Figure 3**). By contrast, increased precipitation reduced the four species diversity indexes in the warming 4°C treatment (**Figure 3**).

### Variations in species asynchrony and community stability

**Figure 4:**
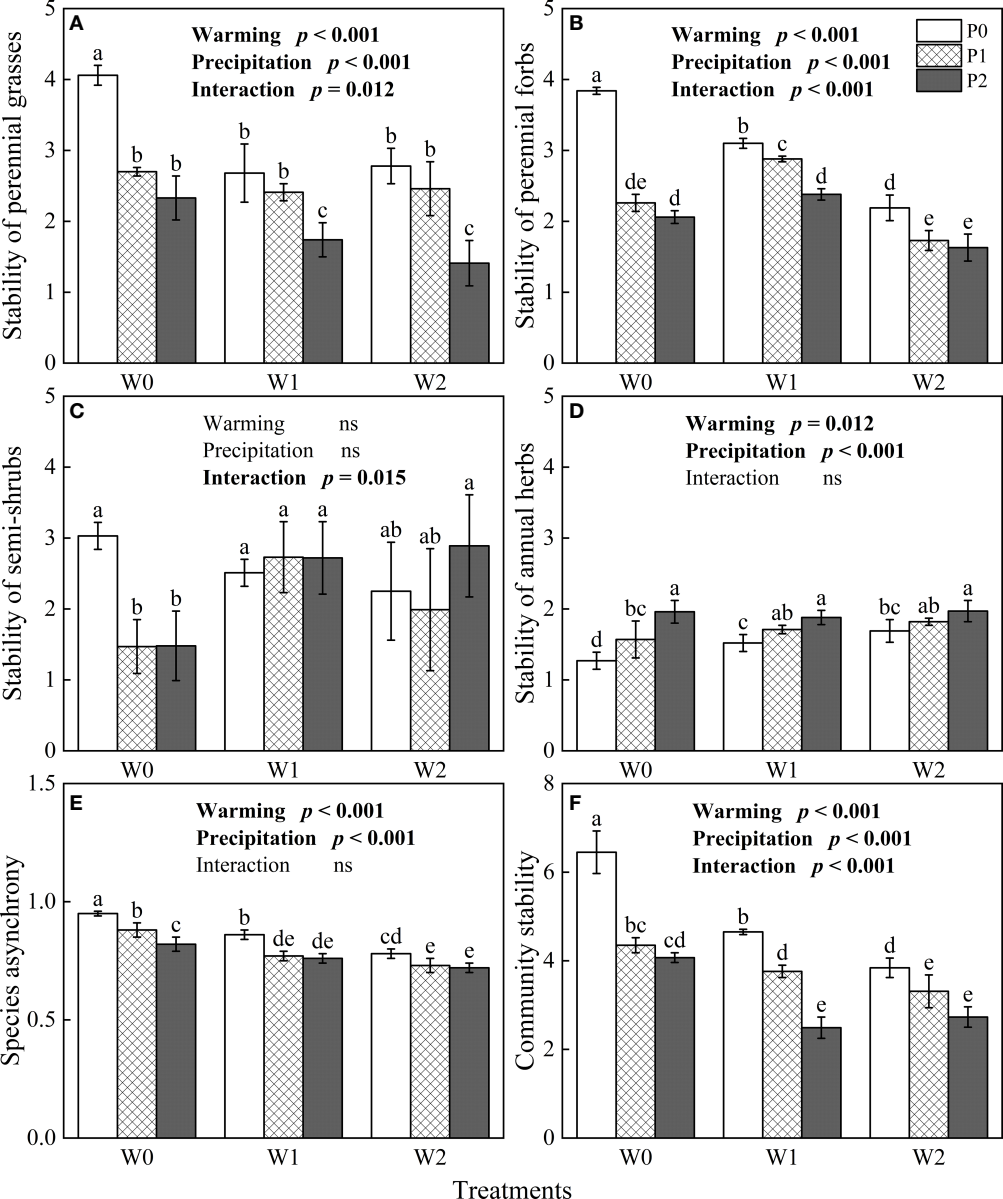
The influences of experimental warming (W), increasing precipitation (P) and their interactions (W×P) on the stability of functional groups **(A–D)**, species asynchrony **(E)**, and community stability **(F)**. W0, W1, W2, P0, P1 and P2 are ambient temperature, ambient temperature increased by 2°C and 4°C, natural precipitation, natural precipitation increased by 25% and 50%, respectively. The same lowercase letters mean insignificant differences between treatments. “ns” is not significant (*P* > 0.05).

The general linear model showed that community stability, species asynchrony and the stability of the four functional groups were significantly influenced by increasing precipitation, experimental warming and their interactions (*P*< 0.05, **Figure 4**). The stability of perennial forbs and perennial grasses, species asynchrony and community stability significantly decreased with warming and increased precipitation (*P*< 0.05, **Figures 4A, B, E, F**). On the contrary, the stability of annual herbs significantly increased with experimental warming and increased precipitation (*P*< 0.05, **Figure 4D**).

### Pearson correlation analysis of community stability and species diversity

**Figure 5:**
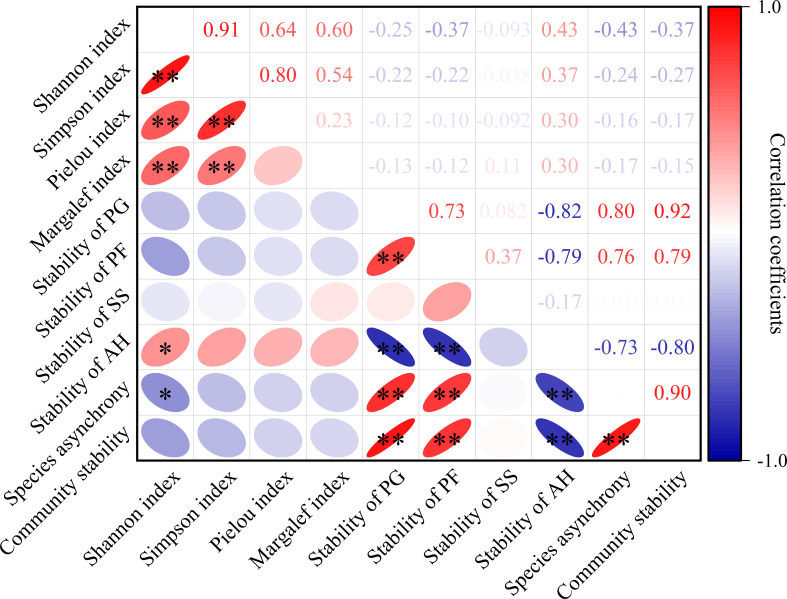
Pearson correlation analysis of species diversity, the stability of four functional groups, species asynchrony and community stability. PG, PF, SS and AH are perennial grasses, perennial forbs, semi-shrubs and annual herbs, respectively. Numbers are correlation coefficients. “*” and “**” are *P*< 0.05 and *P*< 0.01.

Compared with species diversity, species asynchrony and the stability of the four functional groups were significantly related to community stability (*P*< 0.01, **Figure 5**). Community stability was significantly positively correlated with species asynchrony and the stability of perennial forbs and perennial grasses (*P*< 0.01, **Figure 5**), while significantly negatively correlated with the stability of annual herbs (*P*< 0.01, **Figure 5**).

### Structural equation model of climate change on community stability

**Figure 6:**
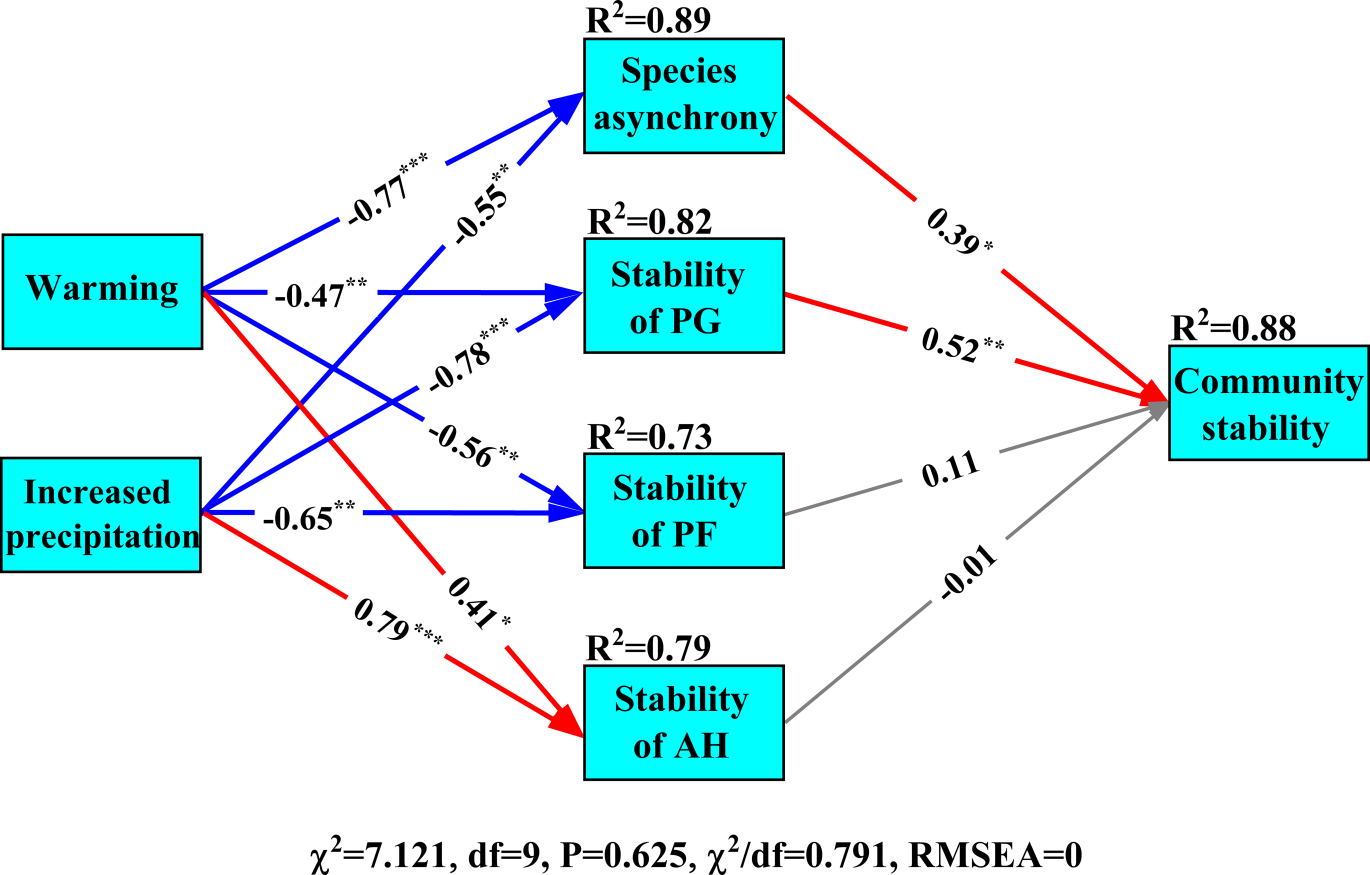
Structural equation model of the influences of experimental climate change on community stability. PG, PF and AH are perennial grasses, perennial forbs and annual herbs, respectively. The red, blue and gray lines indicate significant positive and negative impacts and insignificant impacts, respectively. Numbers are standardized path coefficients. Goodness-of-fit index: χ^2^ is Chi-square, df is degrees of freedom and RMSEA is the root mean square error of approximation. “*, **” and” ***” indicate *P*< 0.05, *P*< 0.01 and *P*< 0.001, respectively.

Results of structural equation modelling showed that experimental warming and increased precipitation reduced the stability of perennial grasses and species asynchrony, which negatively impacted community stability (**Figure 6**). The stability of perennial grasses (path coefficient is 0.52, *P*< 0.01, **Figure 6**) contributed more to community stability than species asynchrony (path coefficient is 0.39, *P*< 0.01, **Figure 6**). Although increased precipitation and experimental warming had significant influences on the stability of annual herbs, the effects on community stability were insignificant (**Figure 6**).

## Discussion

### Variations in species composition have little influence on the species diversity of desert steppe

Experimental warming and increased precipitation had a non-negligible influence on the importance value of four functional groups in our study (**Figure 2**). Studies of the effects of experimental warming and increased precipitation on the species composition of grassland in northern China have commonly found that the proportion of perennial grasses decreased and the proportion of perennial forbs increased in the plant community (Zhang et al., 2015; Tian et al., 2021). Previous studies have concluded that soil water availability (Yang et al., 2011b; Su et al., 2019), soil available nitrogen content (Ren et al., 2017; Tian et al., 2021; Wu et al., 2021) and interspecific competition (Deng et al., 2014; Kuster et al., 2016; Ren et al., 2022) are critical factors in changing community composition. Experimental warming has changed soil available nitrogen content (Wu et al., 2021), which intensified interspecific competition (Ren et al., 2022). Dominant species will change the form of nitrogen obtained to reduce interspecific competition (Kuster et al., 2016). In addition, Ren et al. (2017) considered that long-term increased precipitation intensified the nitrogen limitation of plant community in a temperate grassland of Inner Mongolia. Compared to soil nitrogen content, we consider precipitation as a critical factor regulating species composition in the desert steppe. Under the ambient temperature treatment (W0), increasing precipitation significantly reduced the importance values of perennial grasses and semi-shrubs in our study (**Figures 2A, C**), while significantly increasing the importance values of perennial forbs grasses and annual herbs (**Figures 2B, D**). Perennial grasses, as a functional group with more dominant species in the desert steppe, are very adaptable to arid growing conditions. Increased precipitation has been observed to alter resource allocation among species, resulting in a decrease in the proportion of perennial grasses in the plant community (Lv et al., 2021). By contrast, perennial forbs have high absorption efficiency, and can quickly adapt to the wetter growth environment. This is consistent with the study of Yang et al. (2011b), who concluded that soil water content dominated plant community composition in Inner Mongolian grasslands. Besides the roots of annual herbs are small and shallow in compared with perennial grass. Perennial crops have a more developed roots and lower water productivity with respect to annual species (Giulia and Nathaniel, 2018).

In our study, the influences of increased precipitation and experimental warming on species diversity were insignificant (**Figure 3**), which is consistent with previous research. Zhang et al. (2015) showed that simulated warming insignificantly affected the richness, evenness and diversity of species in the Songnen grassland in Northeast China. Wan et al. (2018) also found that the Simpson index was insignificantly affected by increasing precipitation and warming in Inner Mongolia. We consider that there are two reasons why species diversity in the desert steppe was insensitive to increased precipitation and warming. On the one hand, the desert steppe has few species, with an average of 7.5 species m^-2^ (Wu et al., 2020). The species in the desert steppe have adapted to the low soil moisture and nutrient contents of the habitat, and simulated warming and increased precipitation did not lead to species loss. On the other hand, the OTC equipment used for experimental warming functions as a physical barrier. Although a fan was installed at the bottom of each OTC, the wind velocity inside the OTC is lower than that in the ambient environment, which may seriously affect the distribution of plant seeds. Due to these limitations imposed by the experimental equipment, warming and increased precipitation did not increase the number of species in the OTC. However, some studies have found that increased precipitation improved the richness and diversity of species in the plant community, while warming reduced them (Yang et al., 2011b; Hou et al., 2013). Therefore, the response of species diversity to temperature and precipitation varies between grassland types, and species diversity may be sensitive to temperature and precipitation in grasslands with more species. By contrast, temperature and precipitation insignificantly affects species diversity in grassland types with fewer species.

### The stability of perennial grasses regulated community stability in the desert steppe

Long-term warming and increased precipitation significantly reduced community stability in our study (**Figure 4F**), which is consistent with the results of studies other grasslands of northern China. Wu et al. (2020) suggested that long-term climate warming had a negative impact on community stability in the desert steppe. Yang et al. (2017) also considered that simulated warming reduced the community stability of a temperate steppe in northern China. Early studies suggested that community stability in grasslands was regulated by species diversity (Watts, 1973; Doak et al., 1998), and that ecosystems with complex biodiversity had high stability (Pimm, 1984). In more recent investigations into ecosystem stability, scholars have found that the mechanisms by which species diversity maintains community stability were not applicable to all grassland types. Therefore, the mass ratio theory was proposed by Grime (1998), who suggested that the functional characteristics and diversity of dominant species directly control the primary productivity and stability of grassland. Our study found that long-term simulated warming and increased precipitation reduced the stability of perennial grasses and perennial forbs, species asynchrony and community stability, while increasing the stability of annual herbs (**Figure 4**). In addition, long-term increased precipitation and experimental warming had a negative impact on community stability by reducing species asynchrony and the stability of perennial grasses (**Figure 6**), which were consistent with the mass ratio theory (Grime, 1998). Ma et al. (2017) suggested that warming reduced community stability by reducing species asynchrony, and that community stability also depended on the stability of dominant species in an alpine grassland on the Tibetan Plateau. By contrast, previous studies have shown that the temporal stability of the community was mainly affected by species diversity, and higher species diversity improved the temporal stability of the community (Jiang and Pu, 2009; Gross et al., 2014; Zhang et al., 2018). Our research suggests that these research findings does not apply to the desert steppe in Inner Mongolia. Compared with other grassland types, the desert steppe is a fragile grassland ecosystem with much lower vegetation coverage and species number. Consequently, the contribution of species diversity to community stability is not obvious in the desert steppe of Inner Mongolia. Wu et al. (2020) showed that simulated climate change significantly reduced the stability of dominant species, which was an important reason for the decline of community stability in the desert steppe. In our study, the stability of perennial grasses, as the functional group with the greatest number of dominant species, had an irreplaceable effect on community stability. Therefore, we suggest that community stability depends on the stability of perennial grasses in the desert steppe of Inner Mongolia.

## Conclusion

Experimental warming and increased precipitation, as critical components of climate change, have non-negligible influences on community stability in the desert steppe. Our results showed that increased precipitation and experimental warming significantly altered the plant community composition of the desert steppe, but did not significantly impact species diversity, which supported our first hypothesis. The low number of species in the desert steppe limited the impact of increasing precipitation and warming on species diversity. In addition, we found that simulated warming and increased precipitation had a significant negative impact on community stability by reducing the stability of perennial grasses, which supported our second hypothesis. Community stability in the desert steppe depended on the stability of perennial grasses rather than species diversity. Therefore, we suggest that maintaining a high proportion of perennial grasses in plant communities would be beneficial for the sustainable development of desert steppe under future climate change.

## Data availability statement

The raw data supporting the conclusions of this article will be made available by the authors, without undue reservation.

## Author contributions

GL: Conceptualization, Writing - original draft, Methodology. MH: Data curation, Software. CW and ZW: Formal analysis, Supervision, Writing - review & editing, Funding acquisition. All authors contributed to the article and approved the submitted version.
