# Supplementary material for: The stability of perennial grasses mediates the negative impacts of long-term warming and increasing precipitation on community stability in a desert steppe
Source: Front Plant Sci. 2023 Jul 27;14:1235510. doi: 10.3389/fpls.2023.1235510 (PMC10415016; doi:10.3389/fpls.2023.1235510)
Supplement: Supplementary file 1 [file DataSheet_1.pdf]

Table S1. The physical and chemical properties of the soil (0-30cm) in the study site.

PH value	Soil conductivity (ms m^{-1})	Soil organic matter (g kg^{-1})	Total nitrogen (g kg^{-1})	Total phosphorus (g kg^{-1})	Total potassium (g kg^{-1})	Available nitrogen (mg kg^{-1})	Available phosphorus (mg kg^{-1})	Available potassium (mg kg^{-1})
8.31	153	19.27	1.27	0.31	35.45	74.07	8.61	146.27

Table S2. Species composition and division of plant communities in study site. PG is perennial grasses, PF is perennial forbs, SS is semi-shrubs, AH is annual herbs.

Species name	Functional group	Photosynthesis type
Stipa breviflora Griseb.	PG	C_3
Stipa krylovii Roshev.	PG	C_3
Leymus chinensis (Trin.) Tzvel.	PG	C_3
Cleistogenes songorica (Roshev.) Ohwi.	PG	C_4
Allium mongolicum Regel.	PF	C_3
Convolvulus ammannii Desr.	PF	C_3
Potentilla bifurca L.	PF	C_3
Aster altaicus Willd.	PF	C_3
Iris tectorum Maxim.	PF	C_3
Astragalus galactites Pall.	PF	C_3
Melissilus ruthenicus (L.) Peschkova.	PF	C_3
Allium polyrhizum Turcz. ex Regel.	PF	C_3
Artemisia frigida Willd.	SS	C_3
Kochia prostrata (L.) Schrad.	SS	C_4
Caragana microphylla Lam.	S	C_3
Caragana stenophylla Pojark.	S	C_3
Neopallasia pectinata (Palls) Poljak.	AH	C_3
Artemisia scoparia Waldst. et Kit.	AH	C_3
Chenopodium glaucum L.	AH	C_4
Chenopodium aristatum L.	AH	C_4
Portulaca oleracea L.	AH	C_4
Salsola collina Pall.	AH	C_4

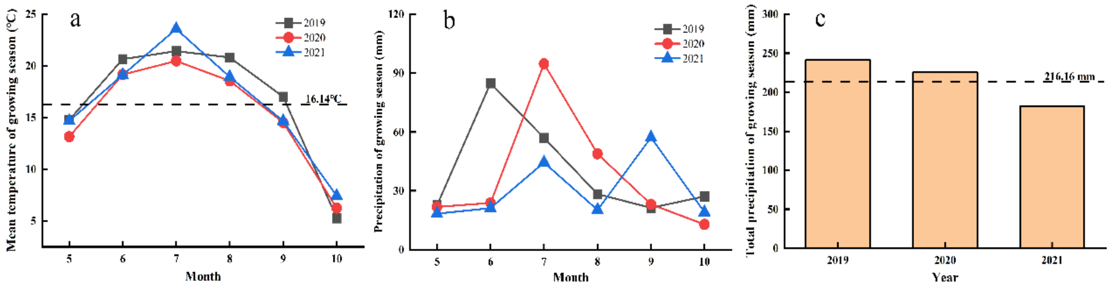

Figure S1. Mean temperature and total precipitation of the plant growing season (May to October) during the 2019-2021. The dotted line indicates the mean temperature and precipitation during the growing season of 2019-2021.

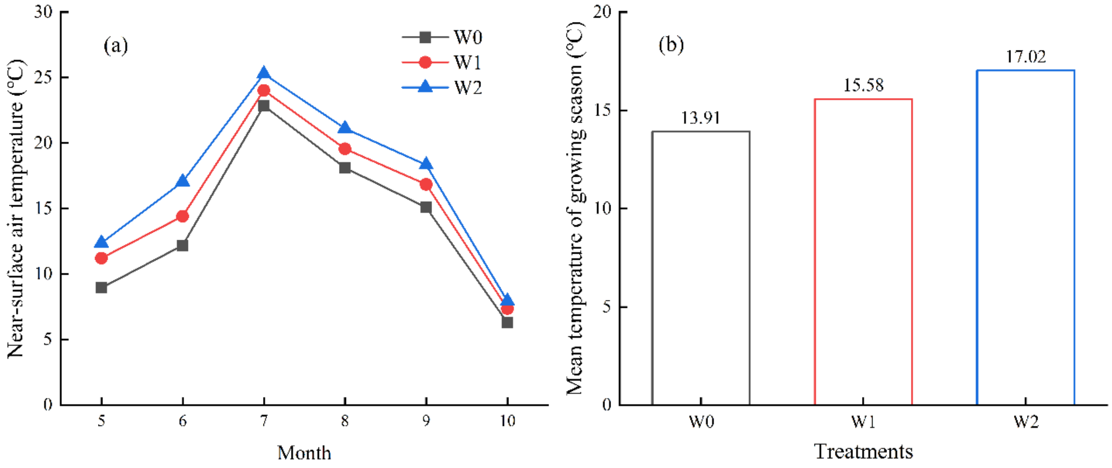

Figure S2. Effects of open top chamber (OTC) on near-surface air temperature during the growing season (May to October) in the 2021.

Calculation formulas for relative frequency, relative density, relative height, relative coverage and relative biomass of species:

In all treatments:

relative frequency (Fr) = Frequency of a species / Sum of frequency of all species

(Eq. 1)

In a treatment:

relative density (Dr) = Density of a species / Sum of density of all species

(Eq. 2)

relative height (Hr) = The average height of a species / The sum of the average heights of all species

(Eq. 3)

relative coverage (Cr) = Coverage of a species / Sum of coverage of all species

(Eq. 4)

relative biomass (Br) = Biomass of a species / Sum of biomass of all species

(Eq. 5)
